# Supplementary material for: Ex Vivo Infection of Human Placental Explants by Trypanosoma cruzi Reveals a microRNA Profile Similar to That Seen in Trophoblast Differentiation
Source: Pathogens. 2022 Mar 16;11(3):361. doi: 10.3390/pathogens11030361 (PMC8952303; doi:10.3390/pathogens11030361)
Supplement: Supplementary file 1 [file pathogens-11-00361-s001.zip › pathogens-1529921-SI.pdf]

pathogens

MDPI

Supplementary data

Supplementary Figure S1

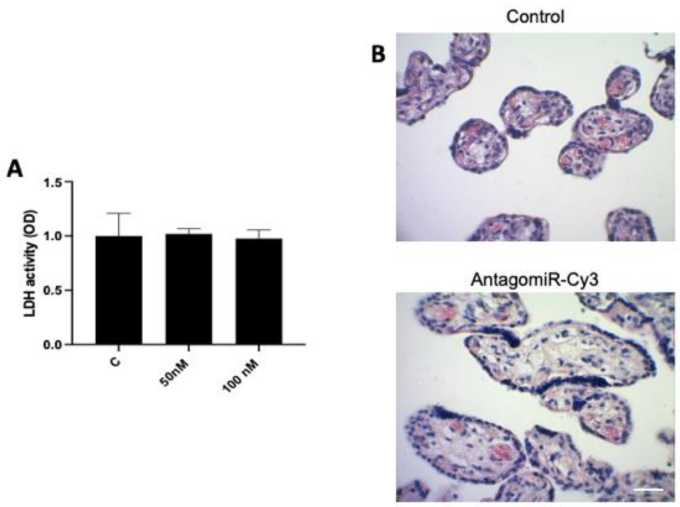

Supplemenary Figure S1. HPE can be effectively transfected without causing tissue damage: HPE were transfected with 50 or 100 nM of AntagomiR-Cy3 during 24 hours. (A) Transfection with either 50 nM or 100 nM does not affect explant viability, as shown by lactate dehydrogenase activity determined by a commercial kit according to manufacturers' instructions. Samples transfected with 100 nM of AntagomiR-Cy3 were processed for routine histological methods and stained with hematoxylin-eosin (B) and showed no evidence of tissue damage. Bar scale: 25 um.
